# Establishment and Validation of an Updated Diagnostic FCM Scoring System Based on Pooled Immunophenotyping in CD34+ Blasts and Its Clinical Significance for Myelodysplastic Syndromes

**DOI:** 10.1371/journal.pone.0088706

**Published:** 2014-02-18

**Authors:** Feng Xu, Xiao Li, Chun-Kang Chang, Juan Guo, Ling-Yun Wu, Qi He, Zheng Zhang, Yang Zhu, Shu-Chen Gu, Wen-Hui Shi, Lu-Xi Song, Ji-Ying Su, Li-Yu Zhou, Xi Zhang, Dong Wu

**Affiliations:** 1 Department of Hematology, Shanghai Jiao Tong University Affiliated Sixth People’s Hospital, Shanghai, China; 2 Department of Internal Medicine, Shanghai Jiao Tong University School of Medicine, Shanghai, China; University of Ottawa, Canada

## Abstract

Abnormal immunophenotypes of hematopoietic cells can be detected by flow cytometry (FCM) to assist the diagnosis of myelodysplastic syndromes (MDS). We previously established a FCM scoring system for the diagnosis of low-grade MDS. In this study, additional valuable antigens were involved in an updated FCM scoring system (u-FCMSS) for all MDS subtypes. The u-FCMSS showed better sensitivity and specificity (89.4% and 96.5%) in distinguishing MDS from non-clonal cytopenia diseases. Validation analysis of u-FCMSS exhibited comparable sensitivity and specificity (86.7% and 93.3%) and high agreement rate (88.9%) of FCM diagnosis with morphological diagnosis at optimal cut-off (score 3). The distribution of FCM scores in different disease stages was also analyzed. The results suggested that early scoring from abnormal expression of mature myeloid/lymphoid antigens and advanced scoring from abnormal expression of stem/progenitor antigens expression constituted the majority of FCM scores of low-grade and high-grade MDS, respectively. High early scoring was generally accompanied by low IPSS-R score and superior survival, whereas high advanced scoring was accompanied by high IPSS-R score and inferior survival. In addition, the low-risk MDS patients with high early scoring and low advanced scoring were revealed as candidates for immunosuppressive therapy, whereas those with high advanced scoring and low early scoring may be more suitable for decitabine treatment. In conclusion, the u-FCMSS is a useful tool for diagnosis, prognosis and treatment selection in MDS. Differences in classes of antigens expressed and in distribution of FCM scores may reflect distinctive stage characteristics of MDS during disease progression.

## Introduction

Myelodysplastic syndromes (MDS) are a class of clonal diseases characterized by abnormal maturation and differentiation of hematopoietic cells, with a high risk of progression to leukemia being observed [1]. MDS is difficult to diagnose due to the complexity and heterogeneity of tumorigenesis. According to WHO criteria, the diagnosis of MDS depends mainly on peripheral cytopenias and morphological changes of hematopoietic cells in bone marrow, as well as other evidences, such as the percentage of ring sideroblasts and abnormal chromosome. However, some MDS patients present with none of the above signs, except peripheral cytopenias. Therefore, we need additional supplemental assays to diagnose MDS. Hematopoietic cells in MDS show various levels of abnormal maturation and differentiation that develop differently from hematopoietic cells in non-clonal cytopenia diseases, and these anomalies can be detected by flow cytometry (FCM). This technique can serve as the auxiliary tool for the diagnosis of MDS [2]–[8].

In our previous study [7], we established a flow cytometric scoring system (FCMSS) to assist the diagnosis of low-grade MDS based on the proportion of CD34+ blasts and co-expressed immunophenotypes such as CD117, CD133, CD15, CD11b, CD4 and CD56. Most patients with low-grade MDS showed high FCM scores because of frequent abnormalities in CD15, CD11b, CD4 and CD56 expression. However, aside from high-grade MDS, some patients with low-grade MDS who may progress rapidly to high-grade MDS did not show frequent abnormality in the expression of mature myeloid/lymphoid immunophenotypes. The FCMSS showed poor diagnostic power in these patients. To improve the diagnostic power of FCM, we need to incorporate other valuable immunophenotypes into the FCMSS to cover the blind area. In addition, the establishment of a universal FCMSS for the diagnosis of all MDS subtypes, including high-grade and low-grade MDS, would provide a quick preliminary screening or comparison with morphologic and clinical diagnosis.

It is widely recognized that MDS show abnormities of the quality and quantity of HSCs. The expression of CD19, CD38 and CD7 on CD34+ cells is considered to be related to differentiation, proliferation and transformation of HSCs [9]–[12]. The percentage of CD34+CD19+ cells (B-cell progenitors) reflects the differentiation from HSCs to B cells [9]. CD34+ cells with low CD38 expression represent early- or low-differentiation HSCs [10]. CD7 expression on CD34+ cells is considered a proliferative and aggressive marker in MDS and leukemia cells [11], [12]. Reductions in the populations of CD34+CD19+ and CD34+CD38+ cells have been used to diagnose MDS independently or in combination with other markers in previous reports [4], [13].

In this study, given the close relationship of CD19, CD38 and CD7 expression with the biological behavior of HSCs, CD19, CD38 and CD7 expression on CD34+ blasts were determined in this study. CD133 was excluded due to its non-specificity for HSCs: it is also expressed in normal endothelial cells and solid cancer cells [14]. Therefore, in this study, we performed a pooled immunophenotyping including stem/progenitor antigens (CD38/CD19/CD117), mature myeloid antigens (CD15/CD11b) and lymphoid antigens (CD4/CD56/CD7) in CD34+ blasts of MDS. Based on these expression pattern, we tried to establish an updated FCMSS to assist in the diagnosis of all MDS subtypes. Meanwhile, the distribution of FCM scores in different stages of the disease and their clinical significance were also investigated.

## Materials and Methods

### Patients

Patients suspected to have MDS (n = 528) during the period from December 2008 to October 2013 who had bi-cytopenias or pan-cytopenias (more than six months) were consecutively enrolled in this study. All clinical and FCM data from these patients were acquired at the first visit. All MDS patients were diagnosed in accordance with the minimum diagnostic criteria established by the Conference on MDS (Vienna, 2006) [15]. The classification and prognostic risk scoring of MDS were performed according to the 2001 WHO criteria and the Revised International Prognostic Scoring System (IPSS-R) [16], [17]. The definition of non-clonal cytopenias diseases is based on clinical characteristics, morphological changes, special biochemical indicators and response to treatment. All suspected MDS patients were diagnosed according to the above criteria. Of the 528 patients, 128 were excluded due to the diagnosis of non-MDS hematological malignancies (leukemia, myeloproliferative diseases, multiple myeloma, lymphoma, etc.). The remaining 400 patients included 270 cases with MDS and 130 cases with non-clonal cytopenia disease, and these patients were divided into two cohorts: a diagnosis cohort and a validation cohort. The diagnosis cohort comprised 180 cases with MDS and 85 cases with non-clonal cytopenia diseases, which are respectively defined as the test group and the baseline group. The validation cohort consisted of 90 cases with MDS and 45 cases with non-clonal cytopenia diseases. All subjects provided written informed consent. The research was approved by the ethics committee of Shanghai Jiao Tong University affiliated Sixth Hospital, and all patient-relevant research strictly abided by the Declaration of Helsinki.

### Ethics Statement

All subjects provided written informed consent. The written informed consent was obtained from patient himself (if minors/children participants, written informed consent was obtained from their guardians). The study was approved by the Ethics Committee of the Sixth Hospital affiliated with Shanghai Jiao Tong University. All patient-relevant research strictly abided by the Declaration of Helsinki.

### Morphological Diagnosis and Cytogenetic Analysis

Bone marrow specimens underwent smear, iron stain and biopsy, followed by immunohistochemical stains when the morphology was difficult to distinguish. Two independent physicians specializing in blood pathology examined each specimen and provided diagnostic reports. At least 500 bone marrow nucleated cells and 200 peripheral blood nucleated cells were counted from each patient. Following the examination of chromosomes, the G-banding technique (Giemsa dyeing) was then used in the analysis of karyotype. For cases in which the G-banding analysis was not available or the number of cells in division was less than 10, FISH analysis was performed to assess 5q-, −7, +8, 20q- and −Y.

### Four-color Flow Cytometry Analysis

The following fluorescent-labeled monoclonal antibodies were used: CD45-PerCP, CD34-APC, CD19-FITC, CD38-PE, CD117-PE, CD7-FITC, CD15-FITC, CD11b-PE, CD4-FITC, CD56-PE and the corresponding isofluorescence controls. All antibodies were purchased from BD Biosciences. Four-color FCM was applied to the analysis of immunophenotypes. The following sets of detection tubes were used: CD45/CD34/CD19/CD38, CD45/CD34/CD7/CD117, CD45/CD34/CD15/CD11b, CD45/CD34/CD4/CD56 and IgG1-PerCP/APC/FITC/PE (isotype control). Heparin anticoagulant marrow solutions were labeled with the relevant antibody set and were then treated with NH_4_Cl (hemolytic reagent). All samples underwent FCM within 4 hours. A flow cytometer (FACS Calibur, Becton Dickinson) equipped with CellQuest software was used for logarithmic (Log) sampling, in which at least 10^5^ total cells and 500 CD34+ cells were acquired and analyzed for most samples. When the percentage of CD34+ cells was low, increasing the number of antibody-labeled cells and prolonging the acquisition time could be used to acquire enough CD34+ cells. CD45/SSC gating was configured to delimit the population of leucocytes; CD34+ blasts with immunophenotypes of CD45^int^CD34^int/high^SSC^low^ were screened to delineated, followed by analysis of the expression of surface immunophenotypes. CD34, CD19, CD38, CD117, CD7, CD15, CD11b, CD4 and CD56 expression in non-clonal cytopenias and low-grade and high-grade MDS are described in Figure S1.

### Establishment of an Updated Flow Cytometric Scoring System (u-FCMSS)

To establish the benchmark for distinguishing MDS patients from non-MDS patients, we used the mean plus or minus 2 standard deviations (SD) or the receiver-operator characteristic (ROC) curve. CD19 expression on CD34+ blasts and the percentage of CD34+ blasts did not show a Gaussian distribution, so we used the ROC curve to determine the benchmark of these immunophenotypes expression. The ROC curve is acquired through the combination of the MDS and baseline groups. The expression of CD38, CD117, CD7, CD15, CD11b, CD4 and CD56 on CD34+ blasts shows a Gaussian distribution, so the mean values of the proportions on CD34+ blasts in the baseline group plus or minus two times the standard deviation were defined as the benchmarks. For CD117, CD7, CD15, CD11b, CD4 and CD56, a sample was defined as abnormality and scored 1 point if any of the tested values was higher than the benchmark. For CD19 and CD38, 1 point was scored if the tested values were lower than the benchmark. In view of the importance of CD34 expression in MDS and the report by Wells et al [2], on the basis of one point for benchmark-5%, one and two additional points, respectively, were given for 5% to 10% and >10% of CD34+ blasts. The points acquired from each abnormality were summed to produce the total score, which was called the FCM score for patients with MDS.

### Validation Analysis of u-FCMSS

The additional validation cohort (n = 135), including 90 patients with MDS and 45 patients with non-clonal cytopenia diseases, was used to validate the u-FCMSS diagnosis. The patients in the validation cohort were diagnosed by morphological observation or u-FCMSS (established based on diagnosis cohort), respectively. Then, the coincidence of the morphological and u-FCMSS diagnoses was compared.

### The Association of FCM Score with WHO Classification, IPSS-R, Transfusion Dependency and Disease Progression in MDS

The relationship of the FCM score with WHO-based prognostic scoring system [18] (MDS-U/RA/RAS, RCMD/RCMD-RS, RAEB-1 and RAEB-2 were defined as score 1, 2, 3 and 4) and transfusion dependency was investigated. Correlation analyses were performed between the FCM score and IPSS-R in 180 cases of MDS and between the FCM score and the three indicators of IPSS-R (cytopenia, marrow blasts, karyotype). The association of the FCM score with survival of MDS was also investigated.

### The Clinical Application of u-FCMSS in Treatment Assessment

26 cases of patients received immunosuppressive therapy (CsA, 3–5 mg/kg/day). Treatment response and FCM score was evaluated after at least 3 months. In 13 cases, patients received lenalidomide treatment (10 mg/m^2^ administered orally daily for three weeks). In 54 cases, patients received decitabine treatment (20 mg/m^2^) administered intravenously over 3 hours daily for 5 days). The treatment course in both agents was repeated every four weeks. Treatment response and FCM score was evaluated every one to two courses. At the evaluation point, the association between the FCM score and treatment response was investigated.

### The Impact of FCM Score on Patients’ Survival in MDS

The FCM score was divided into different categories for analysis of the impact of FCM score on survival. The survival curves were plotted by the Kaplan-Meier method and were compared using the log-rank test. Multivariate or univariate analysis was performed by using the Cox proportional hazard model.

### Statistical Analysis

The chi-square test (R×C) was applied for comparison of the positive rate of immunophenotype expression between different groups. The Mann-Whitney U test was used to compare the differences in flow cytometric score between different groups. Pearson correlation analysis was used for numeric type tests. Spearman correlation analysis was used for ranking correlation tests. *P*<0.05 was considered statistically significant.

## Results

### Patient Characteristics

The baseline group contained a total of 85 patients with non-clonal cytopenias, including 16 patients with iron deficiency anemia, 18 patients with megaloblastic anemia, 15 patients with idiopathic thrombocytopenic purpura, 9 patients with hemolytic anemia, 13 patients with immunopancytopenia, 7 patients with drugs-induced cytopenias, and 7 patients with anemia of chronic diseases. Their median age was 58 years (19–91 years). The test group was composed of 180 MDS patients with a median age of 56 years (15–88 years), including 88 low-grade patients with normal karyotypes, 38 low-grade patients with abnormal karyotypes and 54 high-grade patients, according to WHO classification (RA, RARS, RCMD, RCMD-RS, and MDS-U are defined as low-grade MDS; RAEB-1 and RAEB-2 are defined as high-grade MDS). In the validation group, conventional diagnosis methods confirmed 90 of the 135 patients, with a median age of 55 years (18–92 years), as having MDS; the other patients were diagnosed with non-clonal cytopenias. Details about these MDS patients and their scores are shown in Table 1.

**Table 1:** Clinical and laboratory characteristics of the patients with MDS.

Characteristic	Test group	validation group#
**No. of patients**	180	90
**Sex, no.**		
Male	109	53
Female	71	37
**Median age, y (range)**	56 (15–88)	55 (18–92)
**WHO classification, no.**		
RA/RARS	14	6
RCMD/RCMD-RS	103	50
RAEB-1	30	20
RAEB-2	24	10
MDS-U	9	3
5q syndrome	0	1
**Cytogenetic abnormality, no.**		
Good plus very good	129	63
intermediate	33	21
Poor plus very poor	18	6
**IPSS-R score, no.**		
Low plus very low (≤3.0)	64	26
Int (3.0–4.5)	60	37
High plus very high (>4.5)	56	27
**Immunophenotype expression**		
Median % of CD34+ blasts (range)	1.19 (0.03–26.68)	1.67 (0.16–24.05)
Median % of CD19/CD34 (range)	5.0 (0.3–66.5)	4.6 (0.4–72.2)
Median CD38 RMFI of CD34+ blasts (range)*	343 (28–1535)	430 (28–1089)
Mean % of CD117/CD34 (range)	82 (39–100)	86 (48–100)
Median % of CD7/CD34 (range)	11.2 (0.3–97.9)	13.0 (0.2–93.7)
Median % of CD15/CD34 (range)	25.9 (0.5–89.6)	30.2 (2.5–81.4)
Median % of CD11b/CD34 (range)	29.3 (0.7–95.7)	37.1 (1.8–85.5)
Median % of CD4/CD34 (range)	14.3 (0.3–85.8)	15.7 (0.5–99.1)
Median % of CD56/CD34 (range)	17.4 (0.3–98.7)	14.4 (0.9–98.8)

# Additional validation group included 90 cases with MDS and 45 cases with non-clonal cytopenias. Clinical and laboratory characteristics of MDS patients only were listed in the table.

*RMFI, the mean fluorescence intensity of antigen staining divided by the mean fluorescence intensity of isotype-matched negative control staining.

### Diagnostic Evaluation and Characteristic Analysis of u-FCMSS

In the baseline group, patients with FCM scores of 0–2 and of over 2 points accounted for 96.4% (82/85) and 3.6% (3/85), respectively. In the test group, the occurrence rates for FCM scores of 0–2 and of over 2 (or 3–10) points was 10.6% (19/180) and 89.4% (161/180), respectively, in all patients with MDS (Table 2). For the cut-off of 3 points, the sensitivity and specificity of the updated flow cytometric scoring system (u-FCMSS) to the diagnosis of MDS reached 89.4% (95% confidence interval, 84.1–93.1%) and 96.5% (95% confidence interval, 90.1–98.8%), respectively (Table 3).

**Table 2:** FCM score level of baseline group, low-grade MDS with/without abnormal karyotype and high-grade MDS.

*FCM* *score*	*Baseline* *Group* *(n = 85)*	*MDS group (test group) (n = 180)*					
		Low-grade MDS with normal karyotype(n = 88)		Low-grade MDS with abnormal karyotype(n = 38)		*High-grade MDS*(n = 54)	Total (n = 180)
0	37 (43.5%)	3 (3.4%)		0 (0%)		0 (0%)	3 (1.7%)
1	25 (29.4%)	5 (5.7%)		1 (2.6%)		0 (0%)	6 (3.3%)
2	20 (23.5%)	5 (5.7%)		4 (10.5%)		1 (1.9%)	10 (5.6%)
3	3 (3.6%)	12 (13.6%)		3 (7.9%)		3 (9.3%)	18 (10.0%)
4	0 (0%)	23 (26.1%)		9 (23.7%)		9 (16.7%)	41 (22.3%)
5	0 (0%)	23 (26.1%)		9 (23.7%)		11 (24.1%)	43 (23.9%)
6	0 (0%)	12 (13.6%)		5 (13.2%)		16 (25.9%)	33 (18.3%)
7	0 (0%)	5 (5.7%)		4 (10.5%)		8 (13.0%)	17 (9.4%)
8	0 (0%)	0 (0%)		2 (5.3%)		4 (5.6%)	6 (3.3%)
9	0 (0%)	0 (0%)		1 (2.6%)		1 (1.9%)	2 (1.1%)
10	0 (0%)	0 (0%)		0 (0%)		1 (1.9%)	1 (0.6%)
≥3	3 (3.6%)	75 (85.2%)		33 (86.8%)		53 (98.1%)	161 (89.4%)
median	1	4	5		6		5

Scoring in the Table 2 revealed that most cases from baseline group had FCM score with <3. However, 75 of 88 (85.2%) had FCM score of 3 or higher in low-grade MDS patients with normal karyotype, 33 of 38 (86.8%) had FCM score of 3 or higher in low-grade MDS patients with abnormal karyotype, and 53 of 54 (98.1%) had FCM score of 3 or higher in high-grade MDS.

**Table 3:** Diagnostic sensitivity and specificity of FCM scoring system in patients with MDS.

*FCM* *scores*	*Reference* *group*	*MDS* *group*	*Sensitivity* *	Specificity#
≥0	85	180	100%	0%
≥1	48	177	98.3%	43.5%
≥2	23	171	95.0%	72.9%
≥3	3	161	89.4%	96.5%
≥4	0	143	79.4%	100%
≥5	0	102	56.7%	100%
≥6	0	59	32.8%	100%
≥7	0	26	14.4%	100%
≥8	0	9	5.0%	100%
≥9	0	3	1.7%	100%
≥10	0	1	0.6%	100%

*Sensitivity = positive cases/(positive cases+false negative cases)×100%.

# Specificity = negative cases/(negative cases+false positive cases)×100%.

Distribution analysis of FCM scoring showed that the occurrence rates with FCM scoring 0–2 and over 2 (or 3–10) points were 14.8% (13/88) and 85.2% (75/88) in low-grade patients with normal karyotype, 13.2% (5/38) and 86.8% (33/38) in low-grade patients with abnormal karyotype, and 1.9% (1/54) and 98.1% (53/54) in high-grade patients, respectively. When compared with low-grade patients with abnormal karyotype, low-grade patients with normal karyotype showed a similar occurrence rate of over 2 points. However, the patients with high-grade MDS had a higher occurrence rate of over 2 points and a higher median FCM score compared to low-grade patients (*P*<0.001; *P*<0.001). Details are shown in Table 2.

### Validation Analysis of u-FCMSS in the Second Cohort Including MDS and Non-clonal Cytopenias

As shown in Table 4, the percentage of cases with FCM scores of 0–2 and over 2 points were 60.0% (81/135) and 40.0% (54/135) in the validation group. On the cut-off 3 points, 54 cases of 135 and 81 cases of 135 were diagnosed as non-MDS and MDS by u-FCMSS, respectively. Compared to morphological diagnosis, twelve of 90 patients with MDS weren’t recognized by u-FCMSS; however, in three patients, non-clonal cytopenia was misdiagnosed as MDS by u-FCMSS. The agreement rate between morphological and u-FCMSS diagnosis reached 88.9% at the optimal cut-off (score of 3) (Table 4). Sensitivity and specificity of u-FCMSS acquired by validation analysis is 86.7% and 93.3%, respectively. Initial and validation analyses of u-FCMSS system showed similar sensitivity (88.9% VS 86.7%) and specificity (96.5% VS 93.3%).

**Table 4:** Validation analysis of u-FCMSS in the second cohort including MDS and non-clonal cytopenias diseases.

		*Conventional diagnosis (clinical and morphologic diagnosis)*		
		MDS	Non-clonal cytopenias	Total
**u-FCMSS diagnosis**	MDS	78 (a)	3 (b)	81 (a+b)
	Non-MDS	12 (c)	42 (d)	54 (c+d)
	total	90 (a+c)	45 (b+d)	

Sensitivity = [a/(a+c)]×100% = 86.7%.

Specificity = [d/(b+d)]×100% = 93.3%.

Positive predictive value = [a/(a+b)]×100% = 96.3%.

Negative predictive value = [d/(c+d)]×100% = 77.8%.

Diagnosis agreement rate = [(a+d)/(a+b+c+d)]×100% = 88.9%.

### Distinctive Expression Patterns of Immunophenotypes in Different Disease Stages of MDS

**Figure 1:**
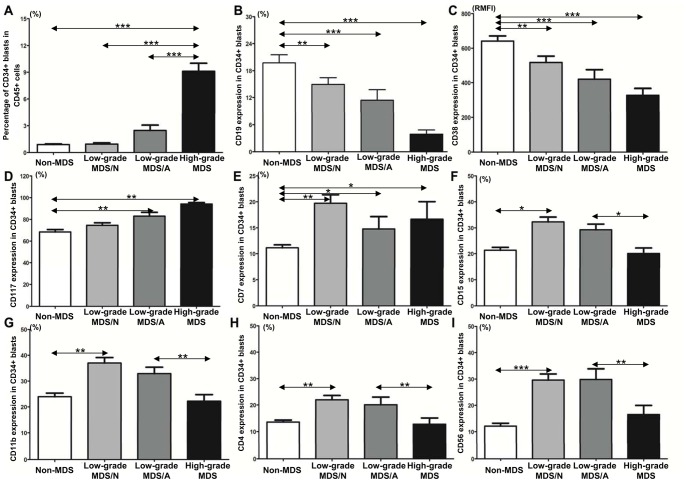
Comparison of immunophenotypes expression in non-clonal cytopenias diseases (non-MDS), low-grade MDS with normal karyotype, low-grade MDS with abnormal karyotype and high-grade MDS. (**A**) Higher percentage of CD34+ blasts is observed in the high-grade MDS than in the low-grade MDS/N, low-grade MDS/A and non-MDS. (**B**) High-grade MDS, low-grade MDS/N and low-grade MDS/A showed lower CD19 expression on CD34+ blasts than non-MDS. High-grade MDS showed lower CD19 expression than low-grade MDS/N and MDS/A. (**C**) Low-grade MDS/N, MDS/A and high-grade MDS showed lower CD38 expression than non-MDS in CD34+ blasts. Obviously, CD38 expression was gradually reduced from low-grade MDS/N to low-grade MDS/A then to high-grade MDS. (**D**) Low-grade MDS/N, MDS/A and high-grade MDS showed higher CD117 expression on CD34+ blasts than non-MDS. CD117 expression was gradually elevated from low-grade MDS/N to low-grade MDS/A then to high-grade MDS. (**E**) Low-grade MDS/N, MDS/A and high-grade MDS showed higher CD7 expression than non-MDS on CD34+ blasts. Both low-grade MDS/N and MDS/A showed higher CD15 (**F**), CD11b (**G**), CD4 (**H**) and CD56 (**I**) expression on CD34+ blasts than high-grade MDS and non-MDS. There is no obvious difference in CD15, CD11b, CD4 and CD56 expression between high-grade MDS and non-MDS. MDS/N, MDS with normal karyotype; MDS/A, MDS with abnormal karyotype. *, *P*<0.05; **, *P*<0.01; ***, *P*<0.001.

As shown in Figure 1 and Table 5, the patients with high-grade MDS showed higher percentages of CD34+ blasts, lower CD19 and CD38 expression, and higher CD117 expression on CD34+ blasts compared with the patients with non-clonal cytopenia (all *P*<0.05). The patients with low-grade MDS also showed lower CD19 and CD38 expression, but they showed higher CD7, CD15, CD11b, CD4 and CD56 expression on CD34+ blasts in comparison to the patients with non-clonal cytopenia (all *P*<0.05). Two distinctive expression patterns of immunophenotypes can be observed: low-grade MDS showed significantly higher CD15, CD11b, CD4 and CD56 expression on CD34+ blasts, whereas high-grade MDS showed remarkably higher CD117 and lower CD19 and CD38 expression on CD34+ blasts as well as a higher percentage of CD34+ blasts. In brief, expression of mature myeloid immunophenotypes and lymphoid immunophenotypes gradually decreased, and stem/progenitor immunophenotypes gradually increased from low-grade MDS with normal karyotype to low-grade MDS with abnormal karyotype and eventually to high-grade MDS.

**Table 5:** Benchmarks from baseline group and the positive case number (over benchmarks) in baseline and MDS group.

*Antigens* *	*Benchmark* *(Cut-off)*	*Baseline group* *(n = 85)*	MDS group (n = 180)			
			*Low-grade with normal Karyotype (n = 88)*	*Low-grade with abnormal Karyotype (n = 38)*	*High-grade MDS (n = 54)*	*P value*
CD34/CD45	1.8%	4 (4.7%)	14 (15.9%)	14 (36.8%)	48 (88.9%)	*P*<0.001
CD19/CD34	7.0%	16 (18.8%)	37 (42.0%)	22 (57.9%)	50 (92.6%)	*P*<0.001
CD38/CD34	364	8 (9.4%)	35 (39.8%)	17 (44.7%)	37 (68.5%)	*P* = 0.003
CD117/CD34	85.0%	15 (17.6%)	37 (42.0%)	24 (63.1%)	50 (92.6%)	*P*<0.001
CD7/CD34	21.2%	3 (3.5%)	29 (33.0%)	7 (18.4%)	13 (24.1%)	*P* = 0.200
CD15/CD34	30.8%	7 (8.2%)	47 (53.4%)	12 (31.6%)	8 (16.7%)	*P*<0.001
CD11b/CD34	35.6%	9 (10.6%)	54 (61.4%)	17 (44.7%)	10 (18.5%)	*P*<0.001
CD4/CD34	21.2%	5 (5.9%)	41 (46.6%)	12 (31.6%)	10 (18.5%)	*P* = 0.003
CD56/CD34	20.7%	6 (7.1%)	56 (63.6%)	18 (47.4%)	14 (25.9%)	*P*<0.001

*CD34/CD45 indicates the proportion of CD34+ blasts in marrow total nucleated cells with CD45 positive.

CD19/CD34, CD117/CD34 and CD7/CD34 represent the expressive proportion of CD19/CD117/CD7 in CD34+ blasts; CD38/CD34 represents RMFI of CD38 expression in CD34+ blasts. For CD34 and CD19, the cut-off values calculated by ROC curve are defined as the benchmark. For CD38, CD117, CD7, CD15, CD11b, CD4 and CD56, the mean value of the proportions of immunophenotypes of the baseline group plus two times the standard deviation (Mean +2SD) was defined as the benchmark. If any of the tested value was higher than the benchmark, it was defined as positive.

### Different Pattern of FCM Scoring Reflects Different Disease Stage of MDS

**Figure 2:**
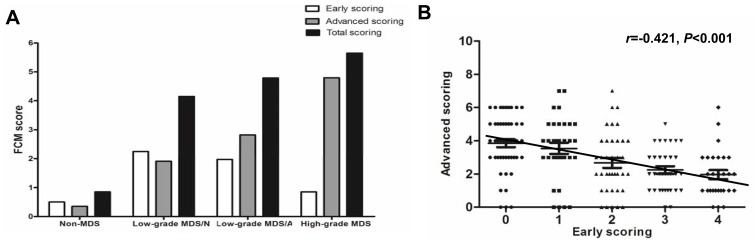
Distinctive distribution of early and advanced scoring in low-grade and high-grade MDS and the relationship between early and advanced scoring. (**A**) Early scoring gradually decreased but advanced scoring gradually increased from low-grade MDS with normal karyotype to low-grade MDS with abnormal karyotype then to high-grade MDS. (**B**) Early scoring showed inversely correlated with advanced scoring (Spearman *r* = −0.363, *P* = 0.001).

FCM scoring from CD34, CD19, CD38, CD117 and CD7 was defined as advanced scoring because these antigens were frequently abnormal in high-grade MDS, and FCM scoring from CD15, CD11b, CD4 and CD56 was defined as early scoring because these antigens were frequently abnormal in low-grade MDS. As shown in Figure 2A, early scoring gradually decreased but advanced scoring gradually increased through the progression from low-grade MDS with normal karyotype to low-grade MDS with abnormal karyotype and then to high-grade MDS (Figure 2A). In addition, early scoring showed an inverse correlation with advanced scoring (Spearman *r* = −0.421, *P*<0.001) (Figure 2B). It seemed that MDS patients could not simultaneously score highly in both early and advanced scoring.

### Relationship of FCM Score to WHO Classification, IPSS-R, Transfusion Dependency and Disease Progression in MDS

**Figure 3:**
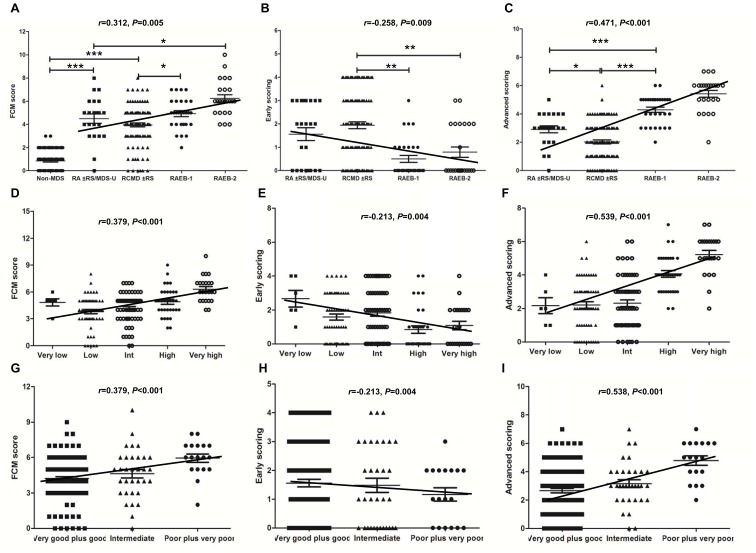
Correlation of FCM score with WHO classification and IPSS-R in MDS. (**A**) The FCM score in RA/RARS/MDS-U, RCMD/RCMD-RS, RAEB-1, and RAEB-2 is significantly increased compared with that in patients with non-clonal cytopenias diseases (all *P*<0.05). FCM score in MDS had a positive correlation with WHO classification (Spearman *r* = 0.312, *P* = 0.005). (**B**) FCM early scoring in RCMD/RCMD-RS is significantly higher than that in RAEB-1 and RAEB-2 (*P* = 0.003; *P* = 0.009). FCM early-scoring in MDS had an inverse correlation with WHO classification (Spearman *r* = −0.258, *P* = 0.009). (**C**) FCM advanced scoring in RAEB-1 or RAEB-2 is significantly higher than that in RA/RARS/MDS-U and RCMD/RCMD-RS (*P*<0.001; *P*<0.001). FCM advanced scoring between each adjacent subgroup differed significantly (all *P*<0.05). FCM advanced-scoring in MDS had a strong positive correlation with WHO classification (Spearman *r* = 0.471, *P*<0.001). (**D**) FCM score had a positive correlation with IPSS-R prognosis classification (Spearman *r* = 0.379, *P*<0.001). (**E**) FCM early scoring in MDS showed reverse correlation with IPSS prognosis classification (Spearman *r* = −0.213, *P* = 0.004). (**F**) FCM advanced scoring in MDS had a strong positive correlation with IPSS prognosis classification (Spearman *r* = 0.539, *P*<0.001). FCM total score (**G**) and advanced scoring (I) showed significantly correlation with cytogenetic prognosis (Spearman *r* = 0.379, *P*<0.001; *r* = 0.538, *P*<0.001), but FCM early scoring (**H**) had a reverse correlation with cytogenetic prognosis (Spearman *r* = −0213, *P* = 0.004). *, *P*<0.05; **, *P*<0.01; ***, *P*<0.001.

The relationship between the FCM score and the different morphologic subgroups is shown in Figures 3A, 3B and 3C. The FCM score in the RA/RARS, RCMD/RCMD-RS, RAEB-1, and RAEB-2 is significantly increased compared with that in the patients with non-clonal cytopenias (Figure 3A). FCM score in MDS had a positive correlation with WHO classification (Spearman *r* = 0.312, *P* = 0.005). Although the FCM score is heterogeneous within each subgroup, FCM early scoring in RCMD/RCMD-RS is significantly higher than in RAEB-1 and RAEB-2 (*P* = 0.003; *P* = 0.009) (Figure 3B). FCM early scoring in MDS had an inverse correlation with WHO classification (Spearman *r* = −0.258, *P* = 0.009). FCM advanced scoring in RAEB-1 is significantly higher than in RCMD/RCMD-RS and RA/RARS (*P*<0.001; *P*<0.001) (Figure 3C). FCM advanced scoring differed significantly between each pair of subgroups (all *P*<0.05). FCM advanced scoring in MDS had a strong positive correlation with WHO classification (Spearman *r* = 0.471, *P*<0.001). In brief, the patients with low-grade MDS showed high early scoring, whereas the patients with high-grade MDS showed high advanced scoring.

**Figure 4:**
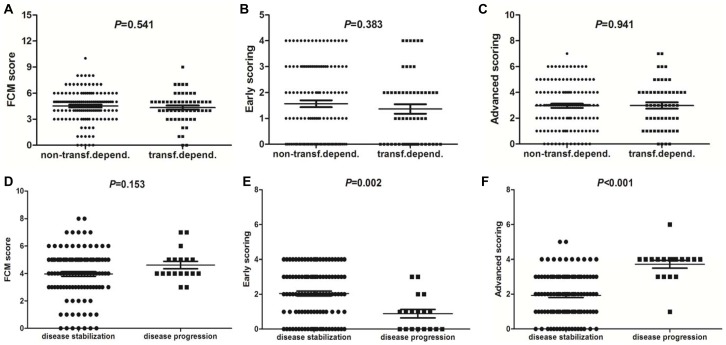
The relationship of FCM score with transfusion dependency and disease progression in MDS. 55 of 180 patients with low-grade MDS were transfusion dependent. There is no obvious difference in FCM total score (**A**), early scoring (**B**) and advanced scoring (**C**) between the patients with and without transfusion dependency. 18 of 126 patients with low-grade MDS progressed towards RAEB-1 or RAEB-2 or AML. There is no obvious difference in FCM total score (**D**) between the patients with and without disease progression (*P* = 0.153). However, the patients with progression toward high-grade MDS or AML showed significantly higher advanced scoring (**F**) but lower early scoring (**E**) than the patients with disease stabilization (*P*<0.001; *P* = 0.002).

In MDS patients, FCM score had a positive correlation with IPSS-R prognosis classification (Spearman *r* = 0.379, *P*<0.001), as seen in Figure 3D. Early scoring in MDS showed an obvious correlation with IPSS prognosis classification (Spearman *r* = −0.213, *P* = 0.004) (Figure 3E). Advanced scoring in MDS had a strong positive correlation with prognosis classification (Spearman *r* = 0.539, *P*<0.001) (Figure 3F). FCM score and advanced scoring showed significant correlation with cytogenetic prognosis (Spearman *r* = 0.379, *P*<0.001; *r* = −0.538, *P*<0.001) (Figure 3G and 3I), but early scoring had an inverse correlation with cytogenetic prognosis (Spearman *r* = −0.213, *P* = 0.004) (Figure 4H).

The patients were also grouped according to transfusion dependency or disease progression. RBC transfusion dependency was defined as having at least one RBC transfusion every 8 weeks over a period of 4 months. Of the 180 patients with MDS, 55 were transfusion dependent. There are no differences in FCM score, early scoring or advanced scoring between the patients with and without transfusion dependency (Figure 4A, 4B and 4C). In addition, 18 of 126 patients with low-grade MDS progressed towards RAEB-1 or RAEB-2 or AML. There is no obvious difference in FCM score between the patients with disease stabilization and disease progression (Figure 4D). However, the patients with disease stabilization showed significant higher early scoring than those with disease progression (*P* = 0.002) (Figure 4E). The patients with progression toward high-grade MDS or AML showed significantly higher advanced scoring than those with disease stabilization (*P*<0.001) (Figure 4F).

### The Role of u-FCMSS in Treatment Selection and Assessment

**Figure 5:**
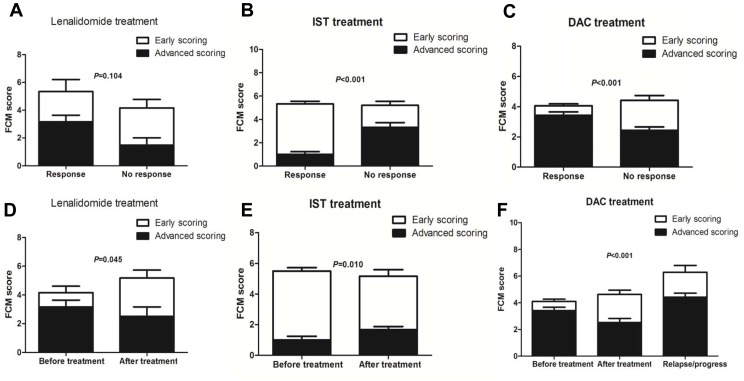
The role of u-FCMSS in treatment selection and assessment. (**A**) The MDS patients with treatment response to lenalidomide have relative high advanced scoring than those without treatment response although no significant difference was observed. (**B**) The MDS patients with treatment response to immunosuppressive therapy (IST) have obvious higher early scoring and lower advanced scoring than those without treatment response before IST (*P*<0.001). (**C**) The MDS patients with treatment response to decitabine (DAC) have obvious higher advanced scoring and lower early scoring than those without treatment response (*P*<0.001). However, the total FCM score have no effect on the treatment response to lenalidomide, IST or DAC. (**D**) In the patients with treatment response to lenalidomide, the advanced scoring decreased slightly, but the early scoring increased obviously after lenalidomide treatment (*P* = 0.045). (**E**) The patients with treatment response to IST showed increased advanced scoring and decreased early scoring after IST (*P* = 0.010). (**F**) The patients with treatment response to DAC showed increased early scoring and decreased advanced scoring after DAC treatment (*P*<0.001). Besides, these patients showed higher advanced scoring and total FCM score when disease relapse.

To analyze role of u-FCMSS in treatment options, the association between the FCM score before treatment and clinical treatment response were investigated. Of the 13 patients who received lenalidomide treatment, 6 cases achieved treatment response (5 major response and 1 minor response). The MDS patients with treatment response had relatively high advanced scoring compared with those without treatment response, although no significant difference was observed (Figure 5A). Of 26 low-risk patients receiving IST, 20 achieved hematologic improvement. The patients with treatment response to IST had obvious higher early scoring and lower advanced scoring than those without treatment response before IST (*P*<0.001) (Figure 5B). High early scoring and low advanced scoring may be indicators of IST in low-risk MDS. In addition, 54 patients received decitabine treatment; 32 responded and 22 did not. The responders have obvious higher advanced scoring and lower early scoring than those non-responders (*P*<0.001) (Figure 5C). High advanced scoring and low early scoring may be an indicator for DAC treatment. However, the total FCM score has no effect on the treatment response to lenalidomide, IST or DAC.

We also investigated the difference in FCM score before and after treatment. In the patients responsive to lenalidomide treatment, the advanced scoring decreased slightly, but the early scoring increased markedly after lenalidomide treatment (*P*<0.001) (Figure 5D). The patients responsive to IST treatment showed increased advanced scoring and decreased early scoring after IST (*P* = 0.010) (Figure 5E). The patients responsive to DAC treatment showed increased early scoring and decreased advanced scoring after DAC treatment (*P*<0.001). Additionally, these patients showed higher advanced scoring and total FCM score upon disease relapse (Figure 5F). The pattern conversion of early and advanced scoring may reflect the treatment effect. Interestingly, the total FCM score did not obviously change, even though the MDS patients showed treatment response to lenalidomide, IST or DAC, indicating that the stemness and clonality of MDS may not be eliminated by these treatments.

### The Effect of FCM Score on Patients’ Survival in MDS

**Figure 6:**
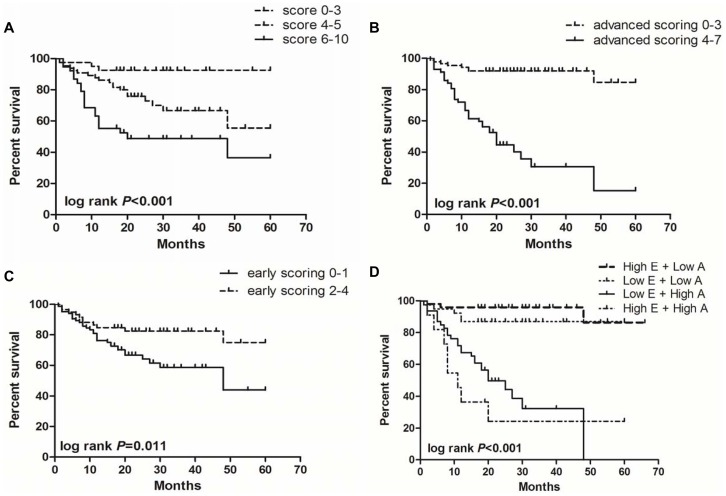
The impact of FCM score on patients survival in MDS. (**A**) The MDS patients with low FCM score had a longer survival, whereas the group with high FCM score had a shorter survival (log rank *P*<0.001). (**B**) The MDS patients with low advanced scoring (score 0–3) had a longer survival, whereas the patients with high advanced scoring (score 4–7) had a shorter survival (log rank *P*<0.001). (**C**) On the contrary, the MDS patients with high early scoring (score 2–4) had a longer survival, whereas the patients with low early scoring (score 0–1) had a shorter survival (log rank *P* = 0.011). (**D**) Interestingly, the MDS patients with high early scoring and high advanced scoring had the worst survival (log rank *P*<0.001).

To analyze the effect of FCM score on survival, the FCM score was divided into three categories: low score (score 0–3), medium score (score 4–5) and high score (score 6–10). The log-rank test showed that survival is significantly different among these three groups (log rank *P*<0.001; Figure 6A). The patients in the group with low scores had longer median overall survival (OS) than those with high scores. Similarly, the median OS was significantly longer in the group with low advanced scoring (score 0–3) than in the group with high advanced scoring (score 4–7) (log rank *P*<0.001; Figure 6B). However, the patients with high early scoring (score 2–4) showed longer median OS than those with low early scoring (score 0–1) (log rank *P* = 0.011; Figure 6C).

In addition, some patients showed simultaneously high early scoring and advanced scoring although early and advanced scoring were mutually exclusive. To analyze the effect of FCM score on the survival of this class of patients, FCM scores were divided into four categories: high early scoring (score 2–4) plus low advanced scoring (score 0–3), low early scoring (score 0–1) plus low advanced scoring (score 0–3), low early scoring (score 0–1) plus high advanced scoring (score 4–7), and high early scoring (score 2–4) plus high advanced scoring (score 4–7). The patients with high early scoring and high advanced scoring had the worst median OS (log rank *P*<0.001; Figure 6D), which suggested that co-expression of mature myeloid/lymphoid immunophenotypes and stem/progenitor immunophenotypes may predict very poor prognosis.

Furthermore, we performed multivariate Cox analysis by integrating several potential risk factors including IPSS-R and FCM score (early scoring, advanced scoring and total scoring) (Table 6). Multivariate analysis showed that advanced scoring is an independent prognostic factor for survival in MDS patients (HR = 1.490, *P* = 0.020).

**Table 6:** The impact of FCM scoring on patients’ survival in univariate and multivariate analysis.

**Univariate analysis**			
Early scoring	0.014	0.733	0.573–0.938
Advanced scoring	<0.001	1.993	1.628–2.440
Total scoring	<0.001	1.545	1.288–1.853
**Multivariate analysis, FCM scoring**			
Early scoring	0.771	0.960	0.729–1.264
Advanced scoring	<0.001	2.059	1.524–2.782
Total scoring	NA	NA	NA
**Multivariate analysis, FCM scoring and IPSS-R**			
Early scoring	0.828	0.970	0.734–1.281
Advanced scoring	0.020	1.490	1.064–2.086
Total scoring	NA	NA	NA
IPSS-R	<0.001	1.501	1.220–1.847

## Discussion

Multiple types of abnormal immunophenotyping pattern have been identified by FCM in MDS, forming an experimental basis for FCM diagnosis in MDS [2], [3], [19]–[21]. The 2006 Vienna conference on MDS and the 2008 Amsterdam European Leukemia Net Conference approved FCM analysis as an important auxiliary tool for the diagnosis of MDS [15], [22]. However, selection of suitable antibodies, specimen sources, objects of analysis and analytical approaches remains difficult. Therefore, it is quite necessary to establish an economical and easily standardized FCM diagnostic system based on the selection of immunophenotypes with specific biological significances that could reflect the malignant nature of clonal cells in MDS. In this study, we chose CD34+ blasts for analysis. Abnormal expression of differentiation/proliferation-associated immunophenotypes (CD19, CD38 and CD117), synchronous expression of non-stage specific immunophenotypes (CD15, CD11b) and expression of non-lineage specific immunophenotypes (CD4, CD56, CD7) in CD34+ blasts may reflect the developmental disorder of MDS clonal cells. Therefore, the establishment of the u-FCMSS based on these abnormalities is feasible. Considering that the MDS patients with abnormal karyotype (indisputable MDS) could be an internal control, similar diagnostic sensitivity (85.2% VS 86.8%) between low-grade patients with normal karyotype and abnormal karyotype supported the hypothesis that the u-FCMSS is also highly effective for low-grade MDS. Meanwhile, the FCM score for the patients with high-grade MDS almost crossed the cut-off score. Overall, the u-FCMSS showed high sensitivity (89.4%) and specificity (96.5%) in diagnosing all MDS subtypes. These findings demonstrate that this u-FCMSS is economical, convenient and reliable for the assisted diagnosis of all MDS subtypes.

There are several other flow cytometric scoring systems (FCMSS) established to assist the diagnosis of MDS. Wells et al [2] reported a FCMSS with 54.8% of sensitivity and 100% of specificity in diagnosing MDS. Ogata et al [4] designed a FCMSS to diagnose low-grade MDS based on immunophenotyping in CD34+ cells, which showed 58% of sensitivity and 100% of specificity. However, on the one hand, their clinical application is limited to high antibodies cost and complicated analysis; on the other hand, the application of these systems in prognosis evaluation wasn’t described. Recently, a multi-centers group reported a FCMSS with 69% of sensitivity and 92% of specificity by integrating four parameters: CD34+ myeloblasts, B-progenitor cells, CD45 expression in myeloblasts and granulocyte side scatter value [23]. The bias from inter- laboratories may have negative effect on the diagnostic power. It is still difficult to construct standardized FCM detection assay in different labs. The choice of FCMSS should be considered according to specific conditions and requirements.

Usually, abnormal expression of different immunophenotypes may be caused by different biological features. We speculated that these immunophenotypes might be associated with three pathological features: defective differentiation, excess proliferation and abnormal response to marrow microenvironment of MDS clonal cells. First, low expression of CD19 and CD38 on CD34+ blasts could be observed in MDS patients, which revealed abnormal development of B-cell progenitors and an increased percentage of early or low-differentiation HSCs [13]. Reduced CD19 and CD38 expression on CD34+ blasts represented defective differentiation in MDS clonal cells. Second, high expression of CD117 and CD7 on CD34+ blasts may represent high proliferation of MDS clonal cells and high risk of disease progression. Similar findings have been reported in other studies [11], [24]. Lastly, high CD15, CD11b, CD4 and CD56 expression on CD34+ blasts in patients with low-risk MDS may reflect the heterogeneity of clonal evolution. The patients with low-risk MDS usually have a stronger self-immune surveillance reaction to malignant clonal cells in marrow microenvironment [25], [26]. We speculated that abnormal CD34+ polyclones might compete for survival during early stages of the disease by expressing non-lineage specific immunophenotypes for escaping the monitoring and killing by the autoimmune system. In brief, the biological significance of these immunophenotypes formed a theoretical basis and convincing body of evidence for FCM diagnosis in MDS.

In this study, we found that low-grade MDS showed significant abnormality of CD15, CD11b, CD4 and CD56 expression on CD34+ blasts, whereas high-grade MDS showed significant abnormality of CD19, CD38, CD117 and CD7 expression on CD34+ blasts as well as a higher overall percentage of CD34+ blasts. The setting of early scoring and advanced scoring is performed based on the distinctive expression patterns of immunophenotypes, which is quite helpful for pre-classification of MDS and evaluation of disease stage. The low-grade patients diagnosed by morphological examination who showed high advanced scoring may suggest a high risk of disease progression. However, the high-grade patients diagnosed by morphological examination who showed high early scoring may suggest a low risk of disease progression. In fact, the low-grade patients with progression toward high-grade MDS or AML showed significantly higher advanced scoring. The combination of early scoring, advanced scoring and the percentage of CD34+ blasts could contribute to the diagnosis of some patients who are shifting from RCMD to RAEB-1. Considering that the disease stage is an important component of selecting particular treatments such as immunosuppressive therapy and decitabine treatment [27], [28], the degree of early scoring or advanced scoring may also be helpful for treatment selection. As shown in this study, low-risk MDS patients with high early scoring and low advanced scoring may be candidates for immunosuppressive therapy, whereas those with high advanced scoring and low early scoring may be more suitable for decitabine treatment. Therefore, FCM scores divided into early scoring and advanced scoring could contribute to disease classification, stage evaluation and treatment options for MDS.

The association of FCM score with WHO classification, IPSS and patients’ survival was also analyzed in this study. Inverse correlation of FCM early scoring with WHO classification and strong positive correlation of advanced scoring with WHO classification in MDS patients have confirmed that high early scoring and advanced scoring are important features of low-grade and high-grade MDS, respectively. Positive correlation of FCM score with IPSS-R prognosis was mainly from the contribution of advanced scoring rather than that of early scoring. Univariate survival analysis revealed that high early scoring and advanced scoring predict superior and inferior survival, respectively. Multivariate analysis further confirmed that advanced scoring is an independent prognostic factor for survival in MDS patients. These findings suggested that the FCM score might be a highly valuable prognosis marker for MDS. In addition, we found that the patients with high early scoring and high advanced scoring had the worst survival. Co-expression of mature myeloid/lymphoid and stem/progenitor immunophenotypes, which can also be observed in mixed-phenotype leukemia [29], may show highly disorganized development of clonal cells and suggest high malignancy in MDS.

Taken together, these results revealed an abnormal expression pattern of multiple immunophenotypes, including stem/progenitor, mature myeloid and lymphoid antigens on CD34 blasts in MDS. Based on this pattern, we developed a convenient and economical u-FCMSS diagnostic system with good sensitivity and specificity to assist in the diagnosis of MDS. Meanwhile, the u-FCMSS also has important applications in the classification, progression forecast, prognosis evaluation and treatment selection in MDS.

## Supporting Information

Figure S1 **Immunophenotyping analysis on CD34+ blasts by flow cytometry in non-clonal cytopenias diseases, low-grade and high-grade MDS.** The percentage of CD34+ blasts and the expression of CD19, CD38, CD117, CD7, CD15, CD11b, CD4 and CD56 (from left to right) on CD34+ blasts in non-clonal cytopenias disease (**A**), low-grade MDS (RCMD) (**B**) and high-grade (RAEB-2) (**C**) (from top to bottom) were shown. The expression of CD19, CD38, CD117, CD7, CD15, CD11b, CD4 and CD56 on CD34+ blasts is measured as a percentage. Expression of CD38 in CD34+ blasts is quantified by the relative mean fluorescence intensity (RMFI) (the mean fluorescence intensity of antigen staining divided by the mean fluorescence intensity of isotype-matched negative control staining).
(TIF)
