# Supplementary figures and images for: Establishment and Validation of an Updated Diagnostic FCM Scoring System Based on Pooled Immunophenotyping in CD34+ Blasts and Its Clinical Significance for Myelodysplastic Syndromes
Source: PLoS One. 2014 Feb 18;9(2):e88706. doi: 10.1371/journal.pone.0088706 (PMC3928275; doi:10.1371/journal.pone.0088706)

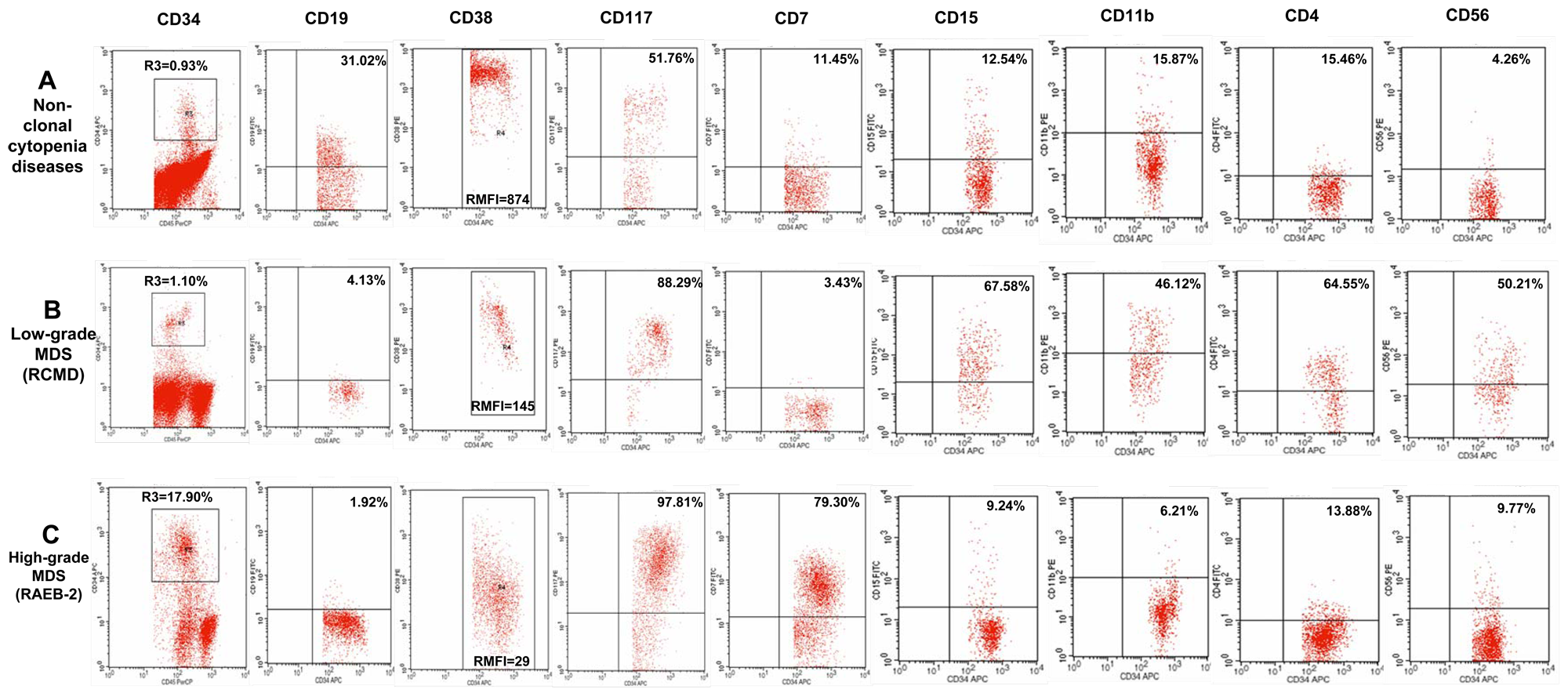

Supplement: Figure S1 — Immunophenotyping analysis on CD34+ blasts by flow cytometry in non-clonal cytopenias diseases, low-grade and high-grade MDS. The percentage of CD34+ blasts and the expression of CD19, CD38, CD117, CD7, CD15, CD11b, CD4 and CD56 (from left to right) on CD34+ blasts in non-clonal cytopenias disease (A), low-grade MDS (RCMD) (B) and high-grade (RAEB-2) (C) (from top to bottom) were shown. The expression of CD19, CD38, CD117, CD7, CD15, CD11b, CD4 and CD56 on CD34+ blasts is measured as a percentage. Expression of CD38 in CD34+ blasts is quantified by the relative mean fluorescence intensity (RMFI) (the mean fluorescence intensity of antigen staining divided by the mean fluorescence intensity of isotype-matched negative control staining). (TIF) [file pone.0088706.s001.tif]
